# Effects of an Innovative High-Fat Diet on Intestinal Structure, Barrier Integrity, and Inflammation in a Zebrafish Model of Visceral Obesity

**DOI:** 10.3390/ijms252312723

**Published:** 2024-11-27

**Authors:** Katarzyna Smolińska, Monika Hułas-Stasiak, Katarzyna Dobrowolska, Jan Sobczyński, Aleksandra Szopa, Ewa Tomaszewska, Siemowit Muszyński, Kacper Smoliński, Piotr Dobrowolski

**Affiliations:** 1 Chronic Wounds Laboratory, Medical University of Lublin, Chodźki St. 7, 20-093 Lublin, Poland; 2 Department of Functional Anatomy and Cytobiology, Maria Curie Sklodowska University, Akademicka St. 19, 20-033 Lublin, Poland; monika.hulas-stasiak@mail.umcs.pl; 3 Faculty of Biology and Biotechnology, Maria Curie Sklodowska University, Akademicka St. 19, 20-033 Lublin, Poland; kasia.dob25@gmail.com; 4 Department of Clinical Pharmacy and Pharmaceutical Care, Medical University of Lublin, Chodźki St. 7, 20-093 Lublin, Poland; jan.sobczynski@umlub.pl (J.S.); aleksandra.szopa@umlub.pl (A.S.); 5 Department of Animal Physiology, Faculty of Veterinary Medicine, University of Life Sciences in Lublin, Akademicka St. 12, 20-950 Lublin, Poland; ewarst@interia.pl; 6 Department of Biophysics, University of Life Sciences in Lublin, Akademicka St. 13, 20-950 Lublin, Poland; siemowit.muszynski@up.lublin.pl; 7 Faculty of Biology, Warsaw University, Żwirki i Wigury St. 101, 02-089 Warsaw, Poland; k.smolinski4@student.uw.edu.pl

**Keywords:** obesity, zebrafish, high-fat diet, intestine, Claudin

## Abstract

High-fat diet (HFD)-induced obesity is a global health concern associated with gastrointestinal disorders. While mammalian models have elucidated the effects of a HFD on intestinal structure and function, its impact on zebrafish, a crucial model for studying diet-induced obesity and gastrointestinal dysfunction, remains inadequately characterized. This study investigated the influence of a HFD on zebrafish intestinal morphology, tight junction (TJ) protein expression, and inflammatory markers. Zebrafish fed a control diet or HFD with 40% or 60% fat exhibited significant alterations in intestinal morphology, with increased villi number but reduced villi width and length, suggesting compensatory responses to dietary stress. TJ protein expression (Claudin 2, Claudin 3, and Claudin 10) showed complex changes, particularly in the HFD60 juvenile group, indicating a multifaceted response in barrier integrity. Pro-inflammatory cytokine IL-6 and TNF-α levels were lower in both the juvenile and adult HFD60 groups than in the HFD40 and control groups, while elevated anti-inflammatory IL-10 levels in HFD60 adult zebrafish suggested activation of compensatory mechanisms. These findings highlight zebrafish as a valuable model for studying the effects of HFD on intestinal health and provide insights into the relationship between dietary fat, gut dysfunction, and inflammation.

## 1. Introduction

Obesity is currently recognized as one of the most significant global health concerns and it places a substantial burden on the healthcare system. In 2024, the NCD Risk Factor Collaboration (NCD-RisC) estimated that over one billion individuals worldwide are currently living with obesity [1,2] (https://www.worldobesity.org/about/about-obesity/prevalence-of-obesity) (accessed on 20 September 2024). The obesity epidemic results from a complex interplay of environmental factors and human behavior, primarily stemming from an imbalance between energy intake and expenditure due to the overconsumption of high-calorie processed foods rich in fats and sugars (junk food) [3]. Overweight and obesity contribute to multiple health problems, including type 2 diabetes, cardiovascular disease, depression, malignancy, and digestive system disorders [4]. Obesity is also associated with chronic gastrointestinal complaints, often overlapping with functional gastrointestinal disorders such as irritable bowel syndrome and dyspepsia [5]. However, the precise mechanisms underlying obesity-associated comorbidities remain unclear. Nonetheless, there is consensus that systemic inflammation is a common factor. Accumulation of adipose tissue in obesity results in the onset of systemic inflammation [6]. The presence of excess macronutrients within adipose tissue results in the release of inflammatory mediators, including tumor necrosis factor-α (TNF-α) and interleukin-6 (IL-6) [7]. These inflammatory cytokines act on the intestinal epithelium, resulting in the weakening of tight junctions (TJ) and an increase in gut permeability, a phenomenon commonly referred to as ‘leaky gut’. Chronic intestinal inflammation that results from obesity further exacerbates this process, ultimately leading to gut microbiota dysbiosis, characterized by an imbalance in gut microbiota, which significantly affects adipose tissue function through several mechanisms. Furthermore, it has been demonstrated that a HFD can also influence the composition of the microbiota, thereby facilitating harmful processes [8,9], supporting the link between gut barrier dysfunction and elevated lipopolysaccharides (LPS) levels, highlighting the critical role of gut health in obesity-related metabolic disturbances.

Consequently, maintenance of gastrointestinal barrier homeostasis is of great importance in the pathogenesis and progression of systemic inflammation, particularly in the context of obesity. The collective actions of epithelial cells, immune cells, nerve cells, and the gut microbiome are responsible for the regulation and maintenance of intestinal barrier integrity. Available evidence suggests that both the structural and functional components of this barrier are altered in individuals with obesity (for a review see [7]). A breach in gut barrier integrity results in metabolic endotoxemia and exacerbation of systemic inflammation, thereby establishing a link between gut dysfunction and obesity-associated comorbidities.

The gastrointestinal tract plays a crucial role in the regulation of food intake by providing signals that modulate appetite-regulating brain areas. These signals include absorbed nutrients, microbial metabolites, and gut hormones, collectively forming a finely tuned system that influences metabolism [10]. The gut’s role as a regulator of appetite and metabolism highlights its potential as a target for therapeutic interventions in obesity [10].

Although established associations between a high-fat diet (HFD) and gastrointestinal disturbances in obesity have been documented, the implications for intestinal barrier integrity and inflammation remain insufficiently explored, particularly in zebrafish models. The exact mechanism by which a HFD alters intestinal morphology and barrier integrity remains unclear. It is essential to elucidate the effects of such eating patterns on intestinal structure and function at both the cellular and molecular levels. To address these knowledge gaps, we hypothesized that a HFD may induce structural changes in the intestines of zebrafish, resulting in altered villi morphology, compromised barrier integrity, and increased inflammatory response. To test this hypothesis, we systematically examined the intestinal histology, TJ protein expression, and inflammatory markers in zebrafish exposed to varying dietary fat levels. This study explored the effects of a novel HFD on gut architecture and inflammatory markers in zebrafish with visceral obesity, focusing on analyzing intestinal histology, histomorphometry, barrier function, and cytokine levels to elucidate the complex alterations in intestinal morphology and inflammatory responses associated with diet-induced obesity.

## 2. Results

**Figure 1:**
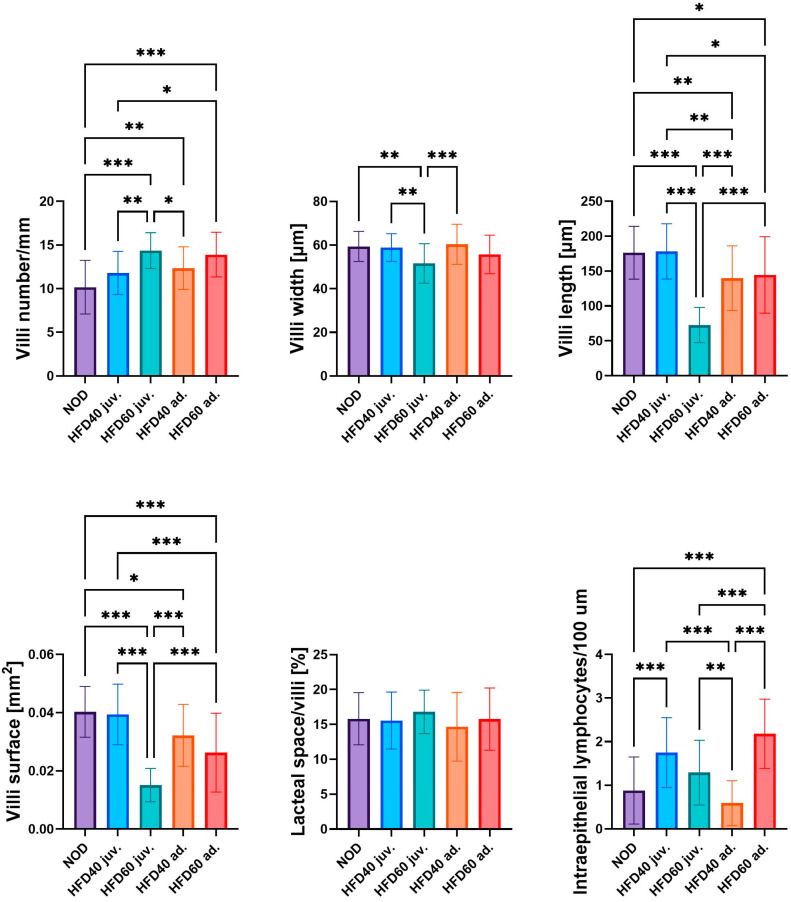
Effect of a high-fat diet (HFD) on intestinal morphology in zebrafish. The figure illustrates the effects of the novel HFD on various aspects of the intestinal morphology in zebrafish. From left to right, the panels show villi number per mm, villi width (μm), villi length (μm), villi surface area (mm^2^), lacteal space per villi (%), and intraepithelial lymphocyte count per 100 μm. The bars (18 weeks of treatment depicted in blue shades and 8 weeks of treatment depicted in red shades) represent the mean values, with the whiskers indicating standard deviations. Statistical comparisons between various groups (NOD, HFD40 juv. (juveniles), HFD60 juv., HFD40 ad. (adults) and HFD60 ad.) are marked, with statistical significance denoted by asterisks: * *p* < 0.05, ** *p* < 0.01, *** *p* < 0.001. These findings demonstrate significant variations in villus morphology and immune response within the intestines of zebrafish under different doses and exposure durations of the HFD.

The influence of a HFD on the selected intestinal structural parameters is shown in Figure 1. All experimental groups were fed a HFD:HFD60 juveniles (juv.) (*p* < 0.001), HFD40 adults (ad.) (*p* < 0.01), and HFD60 ad. (*p* < 0.001), and with the exception of HFD40 juv., demonstrated significantly increased numbers of intestinal villi compared to the control group (NOD). Specimens from the HFD60 juv. group, fed from the second week post-fertilization (juvenile), exhibited the highest number of villi among all groups. Fish fed the HFD60 generally exhibited a higher number of villi than those fed the HFD40; however, the difference was statistically significant only for zebrafish fed from the juvenile stage (*p* < 0.01). Conversely, zebrafish receiving HFD60 ad. demonstrated the narrowest villi, which were significantly narrower than those in the NOD (*p* < 0.01), HFD40 juv. (*p* < 0.01), and HFD40 ad. (*p* < 0.001) groups. This phenomenon was more pronounced with respect to villi length, similar to zebrafish from the HFD60 juv. group exhibited significantly shorter villi than the other groups (*p* < 0.001). No significant differences in villi length were observed between the HFD40 juv. group and the NOD; however, both groups receiving a HFD from the 3rd month post-fertilization demonstrated a significantly reduced villi length compared to the NOD (HFD40 ad. *p* < 0.01 and HFD60 ad. *p* < 0.05). Similar observations were noted regarding villi surface area, which is largely dependent on villi length; however, some differences were more pronounced than others. Zebrafish from the HFD60 juv. group exhibited the smallest villi surface area compared to the other groups (*p* < 0.001). In this instance, greater differences were observed between the NOD and HFD60 ad. groups (*p* < 0.001), whereas fewer differences were noted between the NOD and HFD40 ad groups. (*p* < 0.05), respectively. No statistically significant differences were observed in the proportion of lacteal spaces in the villi between the groups. However, substantial differences were observed in the number of intraepithelial lymphocytes. HFD40 juv. (*p* < 0.001) and HFD60 ad. (*p* < 0.001) exhibited significantly higher numbers of intraepithelial lymphocytes compared to the NOD group. The lowest quantity of intraepithelial lymphocytes was observed in the HFD40 ad group compared with the other HFD groups. Notably, the relationship between the HFD-fed groups was inversely correlated with the duration of HFD exposure. Specifically, zebrafish receiving HFD from the second week post-fertilization demonstrated a higher number of intraepithelial lymphocytes when fed with an additional 40% fat compared to those receiving 60% fat, although this difference was not statistically significant. Conversely, this ratio was inverted in zebrafish receiving a HFD from the 3rd month post-fertilization, when the HFD60 ad. group exhibited a significantly higher number of intraepithelial lymphocytes than the HFD40 ad. (*p* < 0.001).

**Figure 2:**
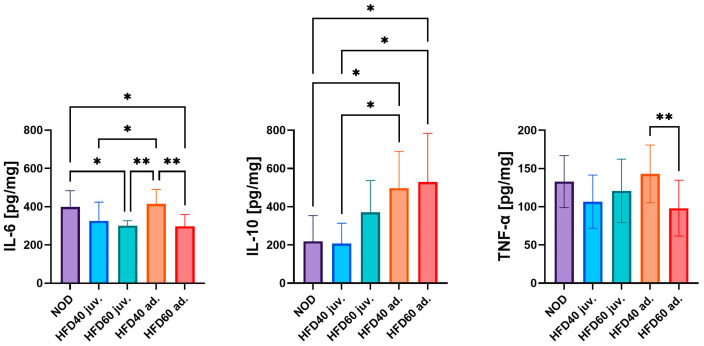
Effects of a high-fat diet (HFD) on pro- and anti-inflammatory cytokine levels in zebrafish intestinal tissue. The figure illustrates the concentrations of pro-inflammatory (IL-6 and TNF-α) and anti-inflammatory (IL-10) cytokines in zebrafish intestinal tissue lysates, as measured by ELISA. The groups were fed NOD (control diet) and HFD40 juv. (juveniles), HFD60 juv., and HFD40 ad. (adults), and HFD60 ad. zebrafish. The bars (18 weeks of treatment depicted in shades of blue and 8 weeks of treatment depicted in shades of red) represent mean cytokine levels (pg/mg), and the whiskers indicate standard deviations. Statistical significance between groups is indicated by asterisks: * *p* < 0.05, ** *p* < 0.01. The results highlight significant changes in cytokine profiles under different HFD conditions, suggesting alterations in both pro- and anti-inflammatory responses due to dietary manipulation.

The levels of pro-inflammatory (TNF-α and IL-6) and anti-inflammatory cytokines (interleukin 10, IL-10) were assessed to determine the impact of a HFD on inflammation in the intestines of zebrafish (Figure 2). The lowest levels of IL-6 were observed in the intestinal samples of zebrafish from the HFD60 juv. and HFD60 ad. compared to the NOD group (*p* < 0.05). Conversely, the highest IL-6 levels were detected in the HFD40 ad. group fed from the third month post-fertilization, with significant differences compared to the other experimental groups, that is, HFD40 juv. (*p* < 0.05), HFD60 juv. (*p* < 0.01) and HFD60 ad. (*p* < 0.01), with the exception of the NOD group. Both HFD40 ad. and HFD60 ad. exhibited the highest level of IL-10 compared to the NOD and HFD40 juv. groups (*p* < 0.05). The only statistically significant difference in TNF-α levels was observed between zebrafish treated with HFD40 ad. and HFD60 ad., where the feed with a higher fat content significantly decreased the level of this cytokine (*p* < 0.01).

**Figure 3:**
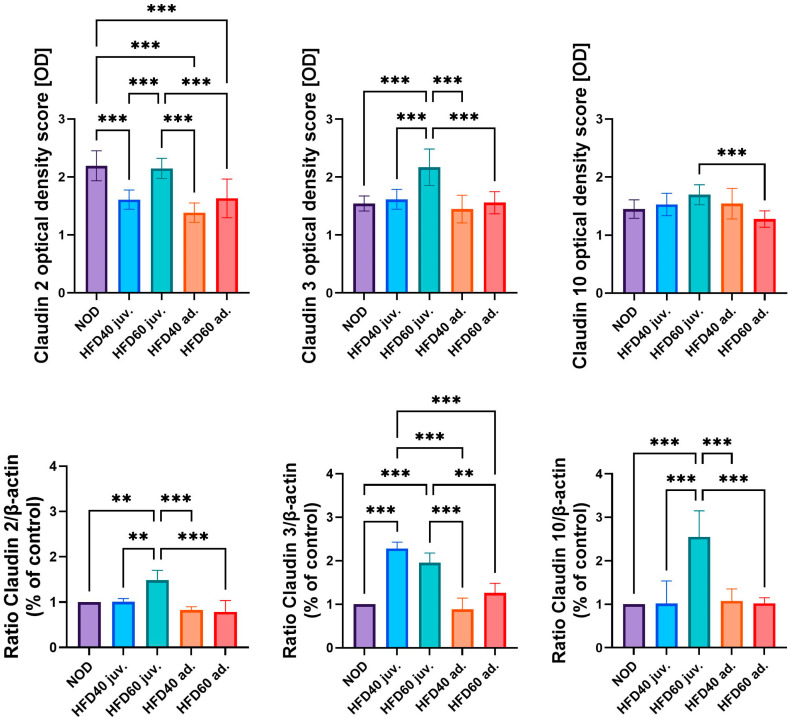
Effects of a high-fat diet (HFD) on tight junction (TJ) proteins in zebrafish intestines. The figure illustrates the effects of a HFD on the expression of TJ proteins in zebrafish intestines, as assessed by immunohistochemistry (IHC) and Western blot (WB). The first row shows the optical density scores from IHC analysis of Claudin 2, Claudin 3, and Claudin 10 measured in different groups: NOD, HFD40 juveniles, HFD60 juveniles, HFD40 adults, and HFD60 adults. The second row presents the WB results, showing the relative expression ratios of Claudin 2, Claudin 3, and Claudin 10 normalized to β-actin (% of control). Bars (18 weeks of treatment depicted in shades of blue and 8 weeks of treatment depicted in shades of red) represent mean values, and whiskers indicate standard deviations. Statistical significance is denoted by asterisks: ** *p* < 0.01, *** *p* < 0.001. These results highlight the significant alterations in TJ protein expression following HFD treatment in zebrafish.

**Figure 4:**
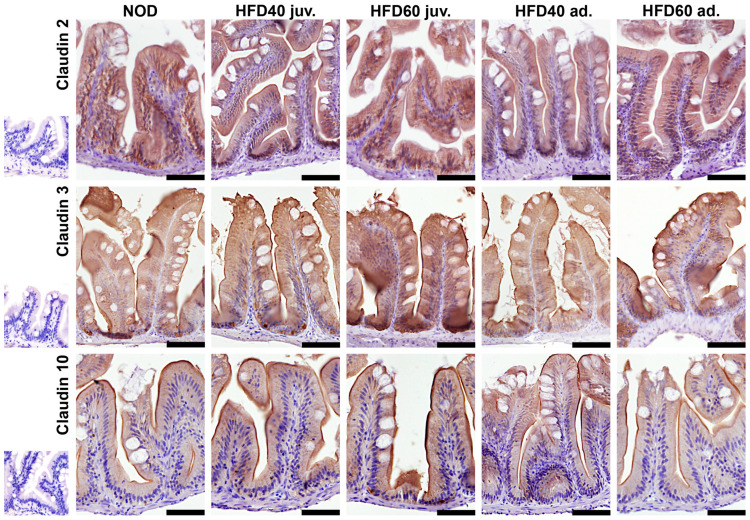
Effect of a high-fat diet (HFD) on the immunolocalization of tight junction (TJ) proteins in zebrafish intestines. The figure illustrates immunohistochemistry (IHC) staining for Claudin 2, Claudin 3, and Claudin 10, showing their immunolocalization and spatial distribution in the TJ of the small intestines of zebrafish by the brown coloration of the epithelium. Zebrafish were categorized as NOD (control) and HFD40 juv. (juveniles), HFD60 juv., and HFD40 ad. (adult), and HFD60 ad. groups. Staining elucidates the localization of these proteins within the intestinal villi, with discernible differences in intensity and distribution across the groups. The black scale bars represent 50 µm, and the inserts on the left depict the antibody controls. The results revealed the effects of different HFD treatments on TJ protein immunolocalization and spatial distribution, with increased staining intensity observed in the HFD groups, particularly in zebrafish receiving a larger dose of fat in the feed from the second week post-fertilization, indicating altered TJ integrity.

**Figure 5:**
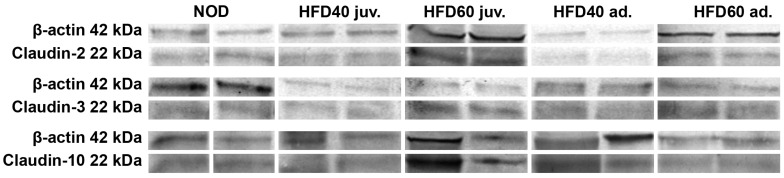
Effect of a high-fat diet (HFD) on the expression of tight junction (TJ) proteins in zebrafish intestines. The figure illustrates representative Western blot (WB) analyses for the expression of TJ proteins Claudin 2, Claudin 3, and Claudin 10 in zebrafish intestines. Zebrafish were grouped into NOD (control) and HFD40 juv. (juveniles), and HFD60 juv. and HFD40 ad. (adults) and HFD60 ad. The protein bands for Claudin 2, Claudin 3, and Claudin 10 (22 kDa) are shown along with β-actin (42 kDa) as a loading control. The blot results highlight the differences in protein expression levels between the groups, with notable upregulation of TJ proteins observed in zebrafish receiving a HFD from the second week post-fertilization, particularly in the HFD60 juv. group, indicating compromised TJ integrity owing to dietary fat intake.

The expression levels of selected TJ proteins, Claudin 2, Claudin 3, and Claudin 10, were analyzed using immunohistochemistry (IHC) and Western blotting (WB), as illustrated in Figure 3 (graphs), Figure 4 (IHC) and Figure 5 (WB). The results of IHC analysis (Figure 3, upper row, and Figure 4) demonstrated significant differences in optical density scores among the experimental groups. Claudin 2 expression was significantly lower in zebrafish treated with HFD40 juv. and the HFD40 ad. and HFD60 ad. groups than in the NOD and HFD60 juv groups (all differences, *p* < 0.001). Claudin 3 in zebrafish from the HFD60 juv. group demonstrated higher expression than that in the other groups (*p* < 0.001). Regarding Claudin 10 expression, only one significant difference was observed: the zebrafish from the HFD60 juv. group exhibited a higher level of this TJ protein than the HFD60 ad. group (*p* < 0.001).

The WB results (Figure 3, bottom row, and Figure 5) mostly corroborated these IHC findings. The relative expression of Claudin 2, normalized to β-actin, was significantly higher in zebrafish from the HFD60 juv. group than in the NOD and HFD40 juv. groups (*p* < 0.01), as well as the HFD40 ad. and HFD60 ad. groups (*p* < 0.001). Similarly, Claudin 3 expression was significantly higher in both groups receiving HFD from the second week post-fertilization, namely HFD40 juv. and HFD60 juv. compared to both the HFD40 ad. and HFD60 ad. groups and the NOD group (*p* < 0.001 in most cases, except HFD60 juv. vs. HFD60 ad. where *p* < 0.01). Claudin 10 expression exhibited an even more pronounced trend, with the highest level observed in the HFD60 juv. group than in the other groups (*p* < 0.001).

## 3. Discussion

Obesity poses a significant threat to global health, necessitating a comprehensive understanding of the mechanisms that regulate body weight to develop effective therapeutic strategies [11]. Food ingestion elicits multiple physiological responses within the gastrointestinal tract, including the secretion of gastrointestinal hormones from enteroendocrine cells that are implicated in appetite regulation [12]. Peptide YY 36 (PYY), glucagon-like peptide 1 (GLP-1), and oxyntomodulin (OXM) are secreted by L-cells located throughout the small intestine. These peptides convey information regarding nutrient availability to the brain and exhibit appetite-suppressing effects [11]. GLP-1 performs various functions, including stimulation of insulin secretion dependent on glucose levels, slowing of stomach emptying, suppression of appetite, enhancement of sodium excretion and urine production, and it influences β-cell growth in rodents. Currently, GLP-1 receptor activators are being extensively used to treat obesity. However, there is a lack of adequate studies examining how a HFD affects the structure of the small intestine, particularly its barrier function, which is directly linked to hormone release and the activity of gut microorganisms in obesity [13].

The results of this study provide insights into the profound impact of a HFD on the intestinal structure and function in a zebrafish model of visceral obesity. Our study builds on previous findings by Smolińska et al. (2024), where the use of an innovative HFD induced visceral obesity in zebrafish, demonstrating the applicability of this model to study obesity-related gastrointestinal alterations [14]. The present study demonstrated that a HFD induced major alterations in the intestinal morphology of zebrafish, particularly affecting the villi structure. Zebrafish subjected to a HFD exhibited an increased number of villi, albeit with reduced width and length, suggesting an imbalance between growth and function. The reduction in villi width and length can directly impair their functionality by decreasing the absorptive surface area available for nutrient uptake, despite their increased number. Smaller and narrower villi provide less surface contact with the luminal contents, leading to reduced nutrient absorption efficiency. Additionally, the structural integrity of shorter and thinner villi may be compromised, affecting their ability to maintain proper barrier function, making them more susceptible to damage or inflammation. Similar findings have been observed in other animal models, where reduced villi dimensions following HFD were associated with malabsorption and disrupted gut homeostasis [15,16,17]. Moreover, even a smaller amount of additional fat in the diet resulted in alterations in intestinal morphology [18]. This compensatory mechanism, wherein villi proliferate to augment the absorptive surface area in response to dietary stress, has been observed in previous studies, which demonstrated that intestinal villi height and structure can be compromised by factors such as weaning stress, pathogens, and dietary alterations [19]. Studies in mice have shown that a HFD can result in a mild reduction in the length of villi in the small intestine, as well as changes in the crypt depth of the colon [20]. Earlier studies demonstrating significant structural alterations in visceral adipose tissue and adipocyte morphology following HFD exposure suggested that analogous adaptive processes related to dietary intervention may extend to the intestinal structures [14]. While the intestine may attempt to compensate by increasing the number of villi, the reduced dimensions of the villi may ultimately compromise their efficiency, reflecting a maladaptive response to prolonged dietary stress [19]. These adaptive changes in the intestine in response to prolonged HFD exposure are in line with previous findings showing compensatory mechanisms such as increased fat deposition and altered absorptive surface area [14]. Analogous to mammalian models, zebrafish fed a HFD exhibited an increased number of villi but reduced villi width and length, suggesting compensatory growth that may be functionally compromised. The observed increase in the number of villi in the HFD60 juv. group aligns with reports of compensatory mechanisms in response to dietary stress, in which the intestine adapts to increased fat intake by expanding absorptive surface area. However, the significant reduction in villi width and length in the same group suggests that the quality of the absorptive surface may be compromised despite this expansion. These findings suggest that dietary lipids may trigger mechanistic pathways that alter the physical properties of intestinal cells, leading to a compromised barrier function. Our findings align with those of previous studies that have demonstrated a significant impact of dietary fatty acids on intestinal cell structure and function. For instance, oleic acid (OA) and palmitic acid (PA), two prominent components of dietary lipids, have been shown to modulate membrane fluidity, mechanosensitive ion channel activity, and the actin cytoskeleton structure in intestinal epithelial cells in vitro. This effect was particularly pronounced in cancerous cells, suggesting differential sensitivity to dietary lipids depending on cell type and physiological state [21]. These insights into mechanotransduction provide a potential mechanistic basis for the structural changes observed in the intestines of HFD-fed zebrafish in the present study. This supports the hypothesis that the structural integrity of the intestine is sensitive to lipid-induced mechanical stress, which can be modulated by specific fatty acids, such as OA and PA. The intestinal effects observed in our zebrafish model are consistent with studies demonstrating that PA, a saturated fatty acid present in high concentrations in the high-fat diet (HFD) formulated for this study, can induce gut permeability and inflammation through the disruption of tight junction (TJ) proteins. These changes contribute to an inflammatory milieu in the intestine mediated by cytokines, such as interleukin 8 (IL-8), as well as the involvement of ceramides, which further exacerbates epithelial barrier dysfunction [22]. Furthermore, as observed in rodent studies, a HFD can exacerbate intestinal permeability, disrupt the epithelial barrier, and increase inflammatory marker expression, rendering zebrafish a relevant model for investigating these processes [7,15]. Moreover, the levels of TJ proteins, specifically pore-forming Claudin 2 and 10 and sealing Claudin 3, were elevated in HFD-fed groups, with the increase dependent on the exposure time, particularly with prolonged exposure to the highest dose, indicating compromised intestinal barrier integrity. These results suggest that a HFD induces an imbalance between these two types of Claudins. Despite a decrease in epithelial Claudin 2 distribution in the two groups receiving 40% additional fat in the diet, the overall levels of Claudin 2, 3, and 10 were significantly elevated in zebrafish from the HFD60 juv. group. However, the total amount of intestinal Claudin 3 was highest in the HFD40 juv. group. Nevertheless, these disturbances in TJs had no effect on the intestinal lacteal spaces.

TJs are critical for maintaining paracellular permeability, and their dysfunction can lead to increased intestinal permeability, also known as ‘leaky gut’ [23]. In this compromised state, the gut allows larger molecules, such as bacterial endotoxins (e.g., LPS), to pass through the epithelial barrier into the bloodstream, triggering systemic inflammation. This heightened permeability not only increases the risk of chronic inflammation but also impairs the ability of the intestine to effectively absorb nutrients, thereby contributing to malabsorption and metabolic disturbances [13]. These findings suggest that disturbances in TJ proteins have far-reaching effects on both gut function and systemic health, amplifying the risk of obesity-related gastrointestinal and metabolic disorders [24]. In addition, these findings align with the observation that visceral fat accumulation induced by an HFD can disrupt intestinal barrier function, as suggested in previous zebrafish studies [14]. Moreover, this also partially aligns with the findings of Gori et al. (2020), who demonstrated that PA, a major component of dietary fats, disrupts TJ integrity, increases intestinal permeability, and contributes to systemic inflammation [25].

In our study, while some HFD groups exhibited heightened levels of inflammatory markers, such as TNF-α, other responses, such as IL-6, varied depending on dietary fat concentration and timing. Specifically, IL-6 levels were significantly reduced in the HFD60 juvenile and adult groups, whereas higher levels of IL-6 were observed in the HFD40 adult group, suggesting a differential response possibly linked to variations in fat content and exposure duration. These findings partially reflect the mechanism proposed by Fang et al. (2022), where PA was shown to induce inflammation in zebrafish intestines through endoplasmic reticulum (ER) stress [26]. Our observations emphasize that while a HFD can trigger inflammatory responses, the extent and type of inflammation may differ based on diet composition, potentially indicating adaptive or compensatory effects at higher fat doses. Additionally, the hepatic steatosis observed in HFD-fed zebrafish further supports the connection between the liver and intestinal inflammation in obesity models [14]. In addition to its effect on TJs, a HFD induces systemic inflammation by disrupting lipid metabolism. In line with our findings, other studies have highlighted the harmful effects of saturated fatty acids such as PA on gut health. PA has been shown to not only increase intestinal permeability but also initiate inflammatory signaling cascades, contributing to liver disease via systemic metabolic disturbances [27]. In parallel with disruptions in intestinal structure and function, dysbiosis induced by a HFD in zebrafish may have direct implications for adipose tissue health. Rosendo-Silva et al. (2023) suggested that gut-derived bacteria and their metabolites, such as LPS, can infiltrate peripheral tissues like adipose tissue, driving local inflammation and contributing to metabolic dysregulation [28]. Collectively, these findings suggest that a HFD leads to intestinal barrier dysfunction and inflammation, which are critical factors in the progression of obesity-related gut and abdominal cavity pathologies.

Additionally, the gut microbiota is increasingly being recognized as a central player in mediating the effects of diet on intestinal health. Dysbiosis driven by a HFD disrupts the intestinal barrier, increases permeability, and contributes to systemic inflammation. Rosendo-Silva et al. (2023) highlighted how dysbiosis caused by a HFD disrupts gut barrier integrity, a phenomenon also reflected in our zebrafish model, in which we observed significant disruptions in both the sealing and pore-forming types of TJ proteins. According to recent findings, these disruptions contribute to endotoxemia, a critical factor driving systemic inflammation and metabolic disturbances [28]. Our findings align with the existing literature that links barrier dysfunction with systemic inflammation, suggesting a similar mechanism in zebrafish fed a HFD. The observed disruption of TJ proteins further supports the hypothesis that intestinal barrier dysfunction contributes to systemic inflammation. Previous studies have shown that such impairments in barrier integrity are linked to the development of chronic inflammatory conditions in obesity [7]. The changes observed in TJ protein expression, particularly in adult zebrafish fed a HFD, are consistent with reports of compromised intestinal barrier function in obese individuals [6]. TJ proteins, including Claudins, play a critical role in maintaining epithelial integrity and their reduction can lead to increased permeability and heightened susceptibility to inflammatory damage. Our findings reinforce the concept that TJ disruption is a key feature of diet-induced intestinal dysfunction, leading to increased paracellular permeability and systemic inflammation.

Fluctuations in pro-inflammatory cytokine levels in HFD-fed zebrafish, particularly IL-6 and TNF-α, mirror findings in human obesity, where gastrointestinal inflammation plays a central role in the development of functional gastrointestinal disorders such as irritable bowel syndrome [29]. This suggests a mechanistic overlap between obesity-induced gut dysfunction in zebrafish and humans, reinforcing the relevance of this model. The inflammatory response observed in our study, characterized by increased intraepithelial lymphocyte infiltration and varying levels of the pro-inflammatory cytokines IL-6 and TNF-α, further underscores the role of chronic inflammation in obesity-related gut dysfunctions. The significant increase in IL-10 levels in the adult zebrafish fed HFD60 and HFD40, however, suggests a potential counter-regulatory response, possibly indicating an attempt by the host to mitigate excessive inflammation, but efficiently only in the HFD40 group, as reflected by low levels of intraepithelial lymphocytes compared to zebrafish from the HFD60 ad group. These findings align with those of Petersen et al. (2022) [10], who reported that obesity triggers both pro- and anti-inflammatory responses, and that the balance between these factors determines the extent of intestinal damage. In addition to barrier dysfunction, leakage of bacterial endotoxins into the circulation is a key consequence of increased intestinal permeability, triggering a systemic inflammatory response. Our findings regarding the modulation of pro-inflammatory cytokines (IL-6 and TNF-α) in zebrafish fed an HFD are consistent with the concept of metabolic endotoxemia discussed by Rosendo-Silva et al. (2023), wherein LPS from gut bacteria translocates into the bloodstream, activates Toll-like receptors, and induces chronic low-grade inflammation [28].

Moreover, the long-term effects of chronic HFD consumption on gut health, including sustained inflammation and barrier dysfunction, are well documented in humans and reflected in the changes observed in our zebrafish model [3]. These findings have important implications for developing therapeutic strategies that target gut health in obesity, particularly through interventions that aim to restore intestinal barrier integrity and modulate gut microbiota. Given the observed changes in TJ proteins in HFD-fed zebrafish, therapeutic approaches that strengthen the intestinal barrier may mitigate the adverse effects of HFD. For instance, treatment with prebiotics, probiotics, or postbiotics can promote beneficial microbial populations, enhance TJ function, and reduce obesity-associated systemic inflammation. The age-dependent effects observed in our study are consistent with previous studies demonstrating that the timing of HFD exposure significantly influences physiological outcomes in zebrafish [14], further underscoring the potential benefit of early dietary interventions to prevent obesity-related gut dysfunction.

Given these findings, our study contributes to a growing body of evidence that highlights the central role of the gut in mediating the effects of dietary fat on obesity. The zebrafish model, owing to its genetic tractability and similarity to human intestinal physiology, is a valuable tool for further exploration of the role of the gut in obesity. The zebrafish model presents several distinct advantages in studying the role of the gut in obesity. Our findings emphasize the central role of the gut in mediating the effects of a HFD on obesity, particularly underlining the possible mechanisms involved in intestinal barrier dysfunction and inflammation. The zebrafish model is an invaluable tool for exploring these processes and offers insights that are highly translatable to human health. Future therapeutic strategies targeting gut health, such as probiotics and dietary interventions, hold promise in mitigating the adverse metabolic consequences of obesity by restoring intestinal integrity and reducing systemic inflammation.

While this study sheds light on the profound effects of HFD on intestinal morphology, barrier integrity, and inflammation, it is important to recognize that it does not encompass the full spectrum of gut responses relevant to metabolic syndrome. Notably, the analysis did not include gut hormone expression or quantification of enteroendocrine cells, which play pivotal roles in the regulation of appetite and systemic metabolic pathways. This limitation was inherent in our preliminary study design, which prioritized foundational data on the structural and inflammatory responses to extreme dietary conditions. Future research will address these gaps by incorporating detailed analyses of gut hormones, enteroendocrine cell distribution, and related gene expression to elucidate the mechanisms linking gut barrier dysfunction to systemic metabolic signaling more comprehensively. Another limitation is that this study did not assess goblet cell numbers or mucin expression, which is another important indicator of gut barrier function. These analyses were not included due to resource constraints but are planned for future studies to provide a more comprehensive understanding of dietary impacts on gut health.

## 4. Materials and Methods

### 4.1. Ethics

This study was approved by the Local Ethics Committee for Animal Experiments at the University of Life Sciences in Lublin, Poland (No. 100/2019). The research was conducted in compliance with the European Union’s regulations on animal protection in scientific studies, as outlined in Directive 2010/63/UE, and implemented in Poland through Legislative Decree 266/2015. The investigation was conducted at the Experimental Medicine Center of the Medical University of Lublin, Poland and adhered to the ARRIVE guidelines.

The 3Rs (replacement, reduction, and refinement) principle was applied in the design of these experiments.

### 4.2. Zebrafish Husbandry

The wild-type AB strain of zebrafish (*Danio rerio*) was obtained from the Experimental Medicine Center at the Medical University of Lublin, Poland (breeder’s license number 077). To minimize stress and prevent uncontrolled sex changes during the experiment, a typical behavioral trait observed in zebrafish, this study employed individuals of both sexes [30].

Fish were maintained in 8 L tanks at a stocking density of 1 L of water per 5 adult individuals. The light cycle was set to 14 h of daylight and 10 h of darkness. The environmental conditions in the quarantine and toxicology room included 15 air changes per hour, 55% humidity, and 22 °C. Water conditions for housing the fish were as follows: temperature 26.0–28.5 °C, daily water exchange rate of 10–20%, pH 6.9–7.5, dissolved oxygen levels above 6 mg/L and nitrogen compound concentrations maintained at NH_4/_NH_3_—0.0 mg/L, NO_2_—0.0–0.1 mg/L and NO_3_—1–5 mg/L. Environmental enrichment was provided by blue tanks and visual elements, such as pads displaying photographs of natural water body substrates or black paint applied beneath the tanks.

### 4.3. High-Fat Diet

A commercially available diet, GEMMA (SKRETTING, Stavanger, Norway), consisting of pellets of various sizes (75, 150, and 300 μm), was used to formulate both the control diet and the experimental HFD. GEMMA constituted 100, 50, and 30% of the NOD, HFD40, and HFD60 feeds, respectively. The experimental HFD featured a modified composition (patent number: Pat. 242859) designed to have a 40% or 60% added beef fat content for HFD40 and HFD60, respectively. This was achieved by incorporating powdered beef fat (GRAU GmbH, Isselburg, Germany), which is composed of 80% fat, 2% ash, 3% protein, and 14% glucose. To improve the stability of the HFD in high-moisture environments, 10% magnesium aluminometasilicate (Neusilin^®^ UFL 2, Fuji Chemical Industries, Tokyo, Japan) was added to the HFD40 and HFD60 formulations. This porous material, which is commonly used in the chemical and pharmaceutical industries, has a high specific surface area (300 m^2^/g) and can absorb approximately 3.2 mL/g of oil and fat [31]. The preparation involved gradually blending the powdered beef fat with the GEMMA pellets, followed by the addition of magnesium aluminometasilicate to the mixture to ensure physical adsorption of the added beef fat onto the Neusilin UFL2 surface as well as its uniform deposition. The types of fats included in the beef fat powder were saturated fats (predominantly palmitic oil glycerides), followed by monounsaturated fats (predominantly oleic acid glycerides). The final fatty acid content in the respective feeds was verified using gas chromatography as previously reported [14].

This detailed formulation process ensures that the HFD is consistent and effective in inducing the desired physiological changes in fish, as demonstrated in previous studies.

### 4.4. Study Design

**Figure 6:**
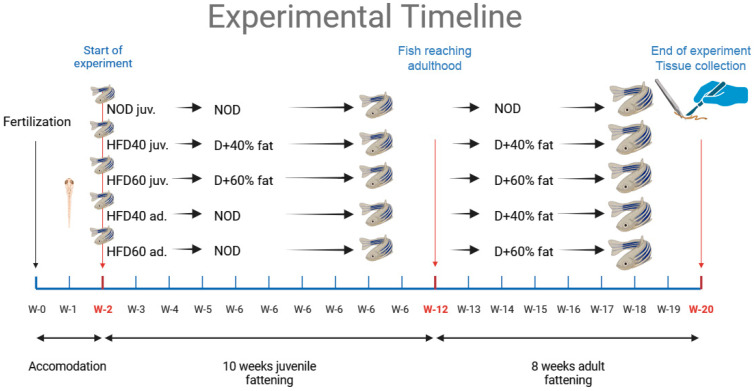
This extensive feeding experiment involved zebrafish receiving five daily meals of their assigned diets along with a daily portion of *Artemia salina*. The research groups included the NOD control set, fed a regular GEMMA diet (SKRETTING, Stavanger, Norway) containing 14% fat; the HFD40 and HFD60 test groups, fed an extra 40% or 60% fat from Beef Fat Powder (GRAU GmbH, Isselburg, Germany); and D+, the standard diet enhanced with the corresponding fat percentage. This study included both the juvenile (juv.), and adult (ad.) zebrafish developmental stages. Image created using BioRender.com. on the basis of a previously published scheme [14].

Two-week-old zebrafish of both sexes were randomly assigned to three dietary groups. Each experimental group included 30 zebrafish, totaling 150 zebrafish in the study. The groups were as follows: control (NOD), HFD40 juveniles, HFD60 juveniles, HFD40 adults, and HFD60 adults. The control group was fed a standard commercial diet, GEMMA, containing 14% fat and supplemented with *Artemia salina*. In parallel, the HFD40 group was fed a newly formulated diet that comprised a commercial basal diet blended with 40% Biff Fat Powder and 10% magnesium aluminometasilicate, along with the simultaneous provision of *Artemia salina*. Similarly, the HFD60 group received a freshly prepared diet consisting of a commercial diet, 60% Biff Fat Powder, 10% magnesium aluminometasilicate, and simultaneous provision of *Artemia salina*. All feeding was administered via calibrated electronic feeders (Eheim AutoFeeder, Deizisau, Germany) with daily checks to verify consistent food portions across groups. Feeders were set to dispense *ad libitum* amounts within a two-minute consumption window per portion, following zebrafish physiological guidelines and established laboratory protocols. Zebrafish were provided with five daily feedings *ad libitum*, and *Artemia salina* was administered once daily. *Artemia salina* was provided daily as a protein supplement, which is especially essential for juvenile zebrafish (up to three months post-fertilization). This supplementation was necessary to ensure adequate protein intake, as the HFD alone did not fully meet the protein requirements of zebrafish. Moreover, *Artemia* served as behavioral enrichment, encouraging natural hunting behaviors vital to zebrafish welfare and development. Subsequently, two groups of zebrafish were fed a diet consisting of a commercial basal diet with simultaneous provision of *Artemia salina* until three months post-fertilization and then switched to a diet with animal fat (either 40% or 60%) and magnesium aluminometasilicate at a concentration of 10%, along with *Artemia salina*, starting at three months post-fertilization. These zebrafish were also fed five times daily, *ad libitum*, using mechanical feeders with *Artemia salina* supplementation once daily. At the end of 20 weeks, the zebrafish were fasted overnight before being measured and weighed, and then euthanized by immersion in Tricaine solution at a concentration of 60 µg/mL for 1 min, as described previously [14]. Intestinal samples were collected from 15 individuals in each group and fixed in buffered formalin (pH 7.0) for subsequent histological and IHC analyses. The intestines of the remaining 15 individuals from each group were immediately frozen in liquid nitrogen for enzyme-linked immunosorbent assays (ELISA) and WB analyses. The study design and timeframe of the experiment are shown in Figure 6, as previously described [14].

### 4.5. Histology Preparation and Histomorphometry Analysis

Small intestine samples were subjected to histological analysis. Tissue samples were fixed in formalin and rinsed under running water for 2 h. Subsequently, the samples were dehydrated in graded ethanol solutions, cleared in xylene, and embedded in paraffin. Cross sections of 4 µm thickness were then obtained using a microtome (HM340E, Microm International GmbH, Walldorf, Germany). The sections were stained using Goldner’s trichrome method to differentiate between the small intestine wall layers and planimetric measurements [32,33]. Microscopic images were obtained from each slide using a bright-field microscope (BX61; Olympus, Tokyo, Japan). Microscopic images of the small intestine were subsequently subjected to histomorphometric analysis using the graphical analysis software ImageJ 1.54 [34]. The following parameters were analyzed: the number of villi per millimeter of mucosa, the length of villi (from the apex to the base), their width (measured at the midpoint of villus length), the surface area of the villi calculated using the modified method of Kisielinski [35], the amount of lacteal spaces as a percentage of villi, and the number of intraepithelial lymphocytes per 100 µm of epithelium [36].

### 4.6. Immunohistochemical Analysis

**Figure 7:**
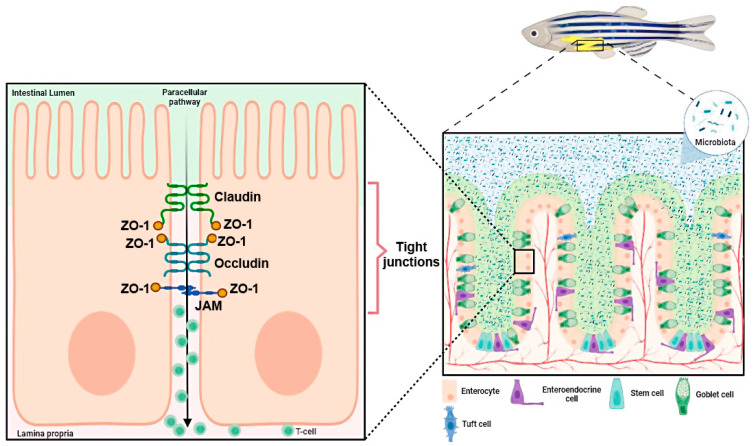
Localization and function of Claudins in the zebrafish intestinal epithelium. This figure illustrates the structure of intestinal epithelial tight junctions (TJ) in zebrafish and highlights the key components involved in regulating paracellular permeability. Claudins, along with other TJ proteins such as Occludin and Junctional Adhesion Molecules (JAM), form a complex network that controls the integrity of the intestinal barrier. Zonula Occludens-1 (ZO-1) links these transmembrane proteins to the actin cytoskeleton, ensuring structural stability. The right panel shows a cross-section of the zebrafish intestine with different selected cell types, including enterocytes, goblet cells, tuft cells, enteroendocrine cells, and stem cells, as well as the microbiota on the luminal surface. These interactions are critical for maintaining intestinal barrier function and preventing pathological conditions, such as inflammation. Image created using BioRender.com.

The immunolocalization of TJ proteins (Claudin 2, 3, and 10), as shown in Figure 7, was evaluated according to a modified protocol previously described [33,37].

Immunohistochemical staining was performed on deparaffinized sections of the small intestine. To mitigate non-specific background staining due to endogenous peroxidase, the slides were incubated in Boxall (Vector Laboratories Inc., Burlingame, CA, USA) for 70 min. The sections were subsequently washed twice with PBS. Heat-induced epitope retrieval was performed in sodium citrate buffer (10 mM sodium citrate, 0.05% Tween 20, pH 6.0) using a pressure cooker (Rapid Cook; Morphy Richards, Swinton, UK). Sections were subsequently cooled to room temperature and washed twice with PBS. Subsequently, the sections were incubated in pre-antibody blocking solution containing 2.5% normal horse serum (Vector Laboratories Inc., Burlingame, CA, USA) for 5 min at room temperature. The sections were then washed twice with phosphate-buffered saline (PBS). The sections were subsequently incubated with primary antibodies for one hour at room temperature in a humidified chamber. All primary antibodies were zebrafish-specific (rabbit as host): anti-Claudin 2, 3, and 10 (51-6100, 34-1700 and 38-8400, respectively; Invitrogen: Thermo Fisher Scientific, Waltham, MA, USA, dilution 1:100). The sections were then washed twice with PBS. Subsequently, the sections were incubated for 30 min with ImmPRESS Horse Anti-Rabbit IgG Polymer Reagent (Vector Laboratories Inc., Burlingame, CA, USA) at room temperature. After two washes in PBS, the reaction was visualized using DAB+ (3,3′-diaminobenzidine chromogen solution and imidazole–HCl buffer (pH 7.5) containing hydrogen peroxide and an antibacterial agent) as a chromogen (DakoCytomation, DakoCytomation Denmark A/S, Glostrup, Denmark) until color development at room temperature. For the control reaction, the primary antibody was replaced with PBS. Counterstaining was performed using Mayer’s hematoxylin (Sigma–Aldrich, Louis, MO, USA) for 30 s. The prepared sections were subsequently dehydrated and coverslipped using DPX (Sigma-Aldrich, Louis, MO, USA). Microscopic images of the small intestine slices from each specimen were subjected to histomorphometry analysis using the graphical analysis software ImageJ 1.54, as previously described.

Using the IHC Profiler [38] ImageJ Plugin with Color Deconvolution and color analysis with the H-DAB model, epithelial integrity was evaluated by comparing pixel brightness values adjusted for DAB staining color detection of each IHC reaction for the analyzed antibodies. The modified H-Score method was employed to calculate the optical density (OD) of the IHC reactions in accordance with the literature [39]. Measurements were conducted exclusively in regions of interest (ROIs) confined to the intestinal epithelium. A semi-automated script was employed to facilitate the measurement process and maintain the reliability [33]. Four measurements were performed for each specimen.

### 4.7. Western Blot Analyses

The small intestines obtained from the experimental groups were homogenized in lysis buffer (125 mM Tris–HCl pH 6.8, 4% SDS, 10% glycerol), boiled in a water bath for 10 min, centrifuged at 10,000× *g* for 10 min, and the supernatant was collected. A Pierce BCA Protein Assay Kit (Thermo Fisher Scientific, Wilmington, DE, USA) was used to determine the protein content. The obtained supernatants were aliquoted and stored at −80 °C. Samples containing 80 µg of protein were separated by 12% SDS–PAGE and then transferred to PVDF membranes (Immobilon-P, Sigma–Aldrich, St. Louis, MO, USA). Following transfer, the membranes were blocked with 3% low-fat milk in PBS for 1 h and incubated overnight at 4 C with primary antibodies: rabbit polyclonal anti-Claudin 2, 3, and 10 antibodies (51-6100, 34-1700 and 38-8400, respectively; Invitrogen: Thermo Fisher Scientific, Waltham, MA, USA, dilution 1:1000). The membranes were washed three times for 10 min with PBS containing 0.05% Triton X-100 (Sigma–Aldrich, St. Louis, MO, USA) and incubated for 2 h with a 1:30 000 dilution of alkaline phosphatase-conjugated goat anti-rabbit IgG (Abcam, Cambridge, UK). The membranes were visualized by adding NBT/BCIP (11681451001, Roche, Basel, Switzerland) alkaline phosphatase substrates, which yielded a blue color. An anti-β-actin antibody (AF7018, Affinity Biosciences, Zhenjiang, China, dilution 1:1000) was used as a loading control. All visible bands were densitometrically quantified and normalized to their corresponding β-actin bands using ImageJ software with the “Gel Analysis” functions. Semi-quantitative analysis was performed for three separately repeated experiments.

### 4.8. Inflammatory Markers Analysis

The concentrations of TNF-α, IL-6, and IL-10 (AffiELISA^®^ Zebrafish TNFa Kit: Ultra-High Sensitivity, AFG-E8345, AffiELISA^®^ Zebrafish IL6 Kit: High Sensitivity, AFG-E4674, and (AffiELISA^®^ Zebrafish IL10 Kit: Ultra-High Sensitivity, AFG-E4452, AffiGEN Inc., Baileys Harbor, WI, USA) were quantified in zebrafish intestine tissue homogenates using commercial zebrafish-specific ELISA kits. Intestinal tissue samples were homogenized, and lysates were prepared for analysis. A Pierce BCA Protein Assay Kit (Thermo Fisher Scientific, Wilmington, DE, USA) was used to determine the protein content. All procedures were performed in accordance with the manufacturer’s protocol. The samples were analyzed in triplicate using a microplate spectrophotometer (BioTek^®^ 800™ TS Absorbance Reader with Gen5™ Secure 3.03 software (BioTek Instruments, Inc., Agilent Technologies, Santa Clara, CA, USA). All ELISA assays showed good reproducibility, with both intra- and inter-assay CVs of less than 10%. The results were calculated using the standard curves generated in the individual assays.

### 4.9. Statistical Analyses

Data are presented as mean ± standard deviation (SD). Differences between means were tested using two-way ANOVA, followed by a post hoc Tukey’s honest significant difference test to correct for multiple comparisons. The statistical model presented below was used to analyze the selected parameters:

x_ij_ = µ + α_i_ + β_j_ + α_i_β_j_ + ε_ijk_

where x_ij_—the observation (body measurements, histomorphometry, IHC, and inflammation parameters), i—the level of the first factor (group: NOD, HFD40, and HFD60), and j—the level of the second factor (treatment length 18 weeks, represented as juv. started in juvenile fish, and 8 weeks represented as ad. started in adult fish), k—the number of measurements, µ—constant (general mean), α_i_—main effect of the first factor, β_j_—main effect of the second factor, α_i_β_j_—interaction, and ε_ijk_—random error.

There were no repeated measurements, as the data presented were derived from randomly selected animals. The normal distribution of the data was confirmed using the Shapiro–Wilk test, and the equality of variance was verified using the Brown–Forsythe test. A two-sided significance level (*p* value) of less than 0.05 was considered statistically significant. GraphPad Prism version 10.2.3, Windows (GraphPad Software, San Diego, CA, USA) was used for all of the statistical analyses.

## 5. Conclusions

This study successfully demonstrated that a HFD significantly affects intestinal morphology, barrier integrity, and inflammatory response in zebrafish, making it an effective model for studying visceral obesity and gastrointestinal dysfunction. The observed increase in villi numbers alongside a reduction in villi width and length suggests compensatory structural changes due to dietary stress, while the disrupted expression of TJ proteins highlights compromised barrier integrity. Elevated levels of pro-inflammatory cytokines, paired with an increase in anti-inflammatory markers, reflect the complex regulatory mechanisms underlying HFD-induced inflammation. These findings underscore the utility of zebrafish as a robust and cost-effective model for exploring obesity-related gut pathologies. Future research should focus on unraveling the molecular pathways involved and assessing potential therapeutic strategies to restore intestinal integrity and manage inflammation in obesity.

The newly prepared HFD40 and HFD60 are described in patent number Pat. 242859.

## Data Availability

Raw data supporting the conclusions of this article will be made available by the authors upon reasonable request.

## References

[1] Bentham J., Di Cesare M., Bilano V., Bixby H., Zhou B., Stevens G.A., Riley L.M., Taddei C., Hajifathalian K., Lu Y. (2017). Worldwide Trends in Body-Mass Index, Underweight, Overweight, and Obesity from 1975 to 2016: A Pooled Analysis of 2416 Population-Based Measurement Studies in 1289 Million Children, Adolescents, and Adults. Lancet.

[2] Phelps N.H., Singleton R.K., Zhou B., Heap R.A., Mishra A., Bennett J.E., Paciorek C.J., Lhoste V.P., Carrillo-Larco R.M., Stevens G.A. (2024). Worldwide Trends in Underweight and Obesity from 1990 to 2022: A Pooled Analysis of 3663 Population-Representative Studies with 222 Million Children, Adolescents, and Adults. Lancet.

[3] Omer T. (2020). The Causes of Obesity: An in-Depth Review. Adv. Obes. Weight Manag. Control.

[4] Apovian C.M., Aronne L.J., Bessesen D.H., McDonnell M.E., Murad M.H., Pagotto U., Ryan D.H., Still C.D. (2015). Pharmacological Management of Obesity: An Endocrine Society Clinical Practice Guideline. J. Clin. Endocrinol. Metab..

[5] Xu G.H., Kim J.A., Kim S.Y., Ryu J.C., Kim Y.S., Jung S.H., Kim M.K., Lee S.H. (2008). Terpenoids and Coumarins Isolated from the Fruits of *Poncirus trifoliata*. Chem. Pharm. Bull..

[6] O’Rourke R.W. (2009). Inflammation in Obesity-Related Diseases. Surgery.

[7] Acciarino A., Diwakarla S., Handreck J., Bergola C., Sahakian L., McQuade R.M. (2024). The Role of the Gastrointestinal Barrier in Obesity-Associated Systemic Inflammation. Obes. Rev..

[8] Serino M., Luche E., Gres S., Baylac A., Bergé M., Cenac C., Waget A., Klopp P., Iacovoni J., Klopp C. (2012). Metabolic Adaptation to a High-Fat Diet Is Associated with a Change in the Gut Microbiota. Gut.

[9] Cani P.D., Bibiloni R., Knauf C., Waget A., Neyrinck A.M., Delzenne N.M., Burcelin R. (2008). Changes in Gut Microbiota Control Metabolic Endotoxemia-Induced Inflammation in High-Fat Diet-Induced Obesity and Diabetes in Mice. Diabetes.

[10] Petersen N., Greiner T.U., Torz L., Bookout A., Gerstenberg M.K., Castorena C.M., Kuhre R.E. (2022). Targeting the Gut in Obesity: Signals from the Inner Surface. Metabolites.

[11] Mok J.K., Makaronidis J.M., Batterham R.L. (2019). The Role of Gut Hormones in Obesity. Curr. Opin. Endocr. Metab. Res..

[12] Farhadipour M., Depoortere I. (2021). The Function of Gastrointestinal Hormones in Obesity—Implications for the Regulation of Energy Intake. Nutrients.

[13] Di Vincenzo F., Del Gaudio A., Petito V., Lopetuso L.R., Scaldaferri F. (2024). Gut Microbiota, Intestinal Permeability, and Systemic Inflammation: A Narrative Review. Intern. Emerg. Med..

[14] Smolińska K., Sobczyński J., Szopa A., Wnorowski A., Tomaszewska E., Muszyński S., Winiarska-Mieczan A., Czernecki T., Bielak A., Dobrowolska K. (2024). Innovative High Fat Diet Establishes a Novel Zebrafish Model for the Study of Visceral Obesity. Sci. Rep..

[15] Rohr M.W., Narasimhulu C.A., Rudeski-Rohr T.A., Parthasarathy S. (2020). Negative Effects of a High-Fat Diet on Intestinal Permeability: A Review. Adv. Nutr..

[16] Walton K.D., Freddo A.M., Wang S., Gumucio D.L. (2016). Generation of Intestinal Surface: An Absorbing Tale. Development.

[17] Grimble G., Geissler C., Powers H. (2017). The physiology of nutrient digestion and absorption. Human Nutrition.

[18] Arias-Jayo N., Abecia L., Alonso-Sáez L., Ramirez-Garcia A., Rodriguez A., Pardo M.A. (2018). High-Fat Diet Consumption Induces Microbiota Dysbiosis and Intestinal Inflammation in Zebrafish. Microb. Ecol..

[19] Szabó C., Kachungwa Lugata J., Ortega A.D.S.V. (2023). Gut Health and Influencing Factors in Pigs. Animals.

[20] Xie Y., Ding F., Di W., Lv Y., Xia F., Sheng Y., Yu J., Ding G. (2020). Impact of a High-Fat Diet on Intestinal Stem Cells and Epithelial Barrier Function in Middle-Aged Female Mice. Mol. Med. Rep..

[21] Bergen J., Karasova M., Bileck A., Pignitter M., Marko D., Gerner C., Del Favero G. (2023). Exposure to Dietary Fatty Acids Oleic and Palmitic Acid Alters Structure and Mechanotransduction of Intestinal Cells in Vitro. Arch. Toxicol..

[22] Ghezzal S., Postal B.G., Quevrain E., Brot L., Seksik P., Leturque A., Thenet S., Carrière V. (2020). Palmitic Acid Damages Gut Epithelium Integrity and Initiates Inflammatory Cytokine Production. Biochim. Biophys. Acta-Mol. Cell Biol. Lipids.

[23] Horowitz A., Chanez-Paredes S.D., Haest X., Turner J.R. (2023). Paracellular Permeability and Tight Junction Regulation in Gut Health and Disease. Nat. Rev. Gastroenterol. Hepatol..

[24] Mishra S.P., Wang B., Jain S., Ding J., Rejeski J., Furdui C.M., Kitzman D.W., Taraphder S., Brechot C., Kumar A. (2023). A Mechanism by Which Gut Microbiota Elevates Permeability and Inflammation in Obese/Diabetic Mice and Human Gut. Gut.

[25] Gori M., Altomare A., Cocca S., Solida E., Ribolsi M., Carotti S., Rainer A., Francesconi M., Morini S., Cicala M. (2020). Palmitic Acid Affects Intestinal Epithelial Barrier Integrity and Permeability in Vitro. Antioxidants.

[26] Fang W., Liu Y., Chen Q., Xu D., Liu Q., Cao X., Hao T., Zhang L., Mai K., Ai Q. (2022). Palmitic Acid Induces Intestinal Lipid Metabolism Disorder, Endoplasmic Reticulum Stress and Inflammation by Affecting Phosphatidylethanolamine Content in Large Yellow Croaker *Larimichthys crocea*. Front. Immunol..

[27] Hanayama M., Yamamoto Y., Utsunomiya H., Yoshida O., Liu S., Mogi M., Matsuura B., Takeshita E., Ikeda Y., Hiasa Y. (2021). The Mechanism of Increased Intestinal Palmitic Acid Absorption and Its Impact on Hepatic Stellate Cell Activation in Nonalcoholic Steatohepatitis. Sci. Rep..

[28] Rosendo-Silva D., Viana S., Carvalho E., Reis F., Matafome P. (2023). Are Gut Dysbiosis, Barrier Disruption, and Endotoxemia Related to Adipose Tissue Dysfunction in Metabolic Disorders? Overview of the Mechanisms Involved. Intern. Emerg. Med..

[29] Ho W., Spiegel B.M.R. (2008). The Relationship between Obesity and Functional Gastrointestinal Disorders: Causation, Association, or Neither?. Gastroenterol. Hepatol..

[30] Westerfield M. (2007). The Zebrafish Book. A Guide for the Laboratory Use of Zebrafish (Danio rerio).

[31] Jadhav N., Pantwalawalkar J., Sawant R., Attar A., Lohar D., Kadane P., Ghadage K. (2022). Development of Progesterone Oily Suspension Using Moringa Oil and Neusilin US2. J. Pharm. Innov..

[32] Suvarna S.K., Layton C., Bancroft J.D. (2019). Bancroft’s Theory and Practice of Histological Techniques.

[33] Iwaniak P., Tomaszewska E., Muszyński S., Marszałek-Grabska M., Pierzynowski S.G., Dobrowolski P. (2022). Dietary Alpha-Ketoglutarate Partially Abolishes Adverse Changes in the Small Intestine after Gastric Bypass Surgery in a Rat Model. Nutrients.

[34] Schindelin J., Arganda-Carreras I., Frise E., Kaynig V., Longair M., Pietzsch T., Preibisch S., Rueden C., Saalfeld S., Schmid B. (2012). Fiji: An Open-Source Platform for Biological-Image Analysis. Nat. Methods.

[35] Kisielinski K., Willis S., Prescher A., Klosterhalfen B., Schumpelick V. (2002). A Simple New Method to Calculate Small Intestine Absorptive Surface in the Rat. Clin. Exp. Med..

[36] Dobrowolski P., Huet P., Karlsson P., Eriksson S., Tomaszewska E., Gawron A., Pierzynowski S.G. (2012). Potato Fiber Protects the Small Intestinal Wall against the Toxic Influence of Acrylamide. Nutrition.

[37] Donaldson J., Świątkiewicz S., Arczewska-Włosek A., Muszyński S., Szymański S. (2021). Modern Hybrid Rye as an Alternative Energy Source for Broiler Chickens Ameliorates Absorption Surface of Initial Segments of Intestines Regardless of Xylanase Supplementation. Animals.

[38] Varghese F., Bukhari A.B., Malhotra R., De A. (2014). IHC Profiler: An Open Source Plugin for the Quantitative Evaluation and Automated Scoring of Immunohistochemistry Images of Human Tissue Samples. PLoS ONE.

[39] Seyed Jafari S.M., Hunger R.E. (2017). IHC Optical Density Score: A New Practical Method for Quantitative Immunohistochemistry Image Analysis. Appl. Immunohistochem. Mol. Morphol..

